# Supplementary material for: Risk assessment in cardiac surgery: Exploring machine learning and laboratory indices as adjunctive tools
Source: PLoS One. 2026 Feb 5;21(2):e0335289. doi: 10.1371/journal.pone.0335289 (PMC12875583; doi:10.1371/journal.pone.0335289)
Supplement: Appendix A Table 4 — We note that multiple procedure codes may be listed per operation. (PDF) [file pone.0335289.s001.pdf]

Appendix A

Table 4. The most common procedures performed in patients in the dataset under consideration. We note that multiple procedure codes may be listed per operation.

Operation	Count
Extracorporeal circulation auxiliary to open heart surgery	3521
Bypass Coronary Artery, One Artery from Left Internal Mammary, Open Approach	2668
Single internal mammary-coronary artery bypass	2157
Excision of Left Saphenous Vein, Percutaneous Endoscopic Approach	1502
Insertion of Infusion Device into Superior Vena Cava, Percutaneous Approach	952
Bypass Coronary Artery, Two Arteries from Aorta with Autologous Venous Tissue, Open Approach	930
Replacement of Aortic Valve with Zooplastic Tissue, Open Approach	892
Excision of Right Saphenous Vein, Percutaneous Endoscopic Approach	866
Open and other replacement of aortic valve with tissue graft	857
(Aorto)coronary bypass of two coronary arteries	788
(Aorto)coronary bypass of three coronary arteries	750
Venous catheterization, not elsewhere classified	725
Bypass Coronary Artery, One Artery from Aorta with Autologous Venous Tissue, Open Approach	641
Bypass Coronary Artery, Three Arteries from Aorta with Autologous Venous Tissue, Open Approach	628
Excision of Left Radial Artery, Percutaneous Endoscopic Approach	553
Bypass Coronary Artery, One Artery from Aorta with Autologous Arterial Tissue, Open Approach	503
(Aorto)coronary bypass of one coronary artery	474
Supplement Mitral Valve with Synthetic Substitute, Open Approach	451
Bypass Coronary Artery, One Artery from Right Internal Mammary, Open Approach	407
Central venous catheter placement with guidance	329
Bypass Coronary Artery, Two Arteries from Left Internal Mammary, Open Approach	324
Excision of Mitral Valve, Open Approach	298
Replacement of Aortic Valve with Synthetic Substitute, Open Approach	284
Open heart valvuloplasty of mitral valve without replacement	284
Replacement of Thoracic Aorta, Ascending/Arch with Synthetic Substitute, Open Approach	262
Destruction of Conduction Mechanism, Open Approach	262
Occlusion of Left Atrial Appendage with Extraluminal Device, Open Approach	261
Open and other replacement of aortic valve	256
(Aorto)coronary bypass of four or more coronary arteries	243
Resection of vessel with replacement, thoracic vessels	233
Left heart cardiac catheterization	196
Insertion of Infusion Device into Right Atrium, Percutaneous Approach	177
Hemodialysis	172
Replacement of Mitral Valve with Zooplastic Tissue, Open Approach	167
Insertion of Monitoring Device into Upper Artery, Percutaneous Approach	164
Insertion of Pacemaker Lead into Right Ventricle, Percutaneous Approach	159
Bypass Coronary Artery, Four or More Arteries from Aorta with Autologous Venous Tissue, Open Approach	152
Arterial catheterization	145
Insertion of Pacemaker Lead into Right Atrium, Percutaneous Approach	145
Supplement Tricuspid Valve with Synthetic Substitute, Open Approach	145
Excision, destruction, or exclusion of left atrial appendage (LAA)	139
Cardioplegia	126
Excision or destruction of other lesion or tissue of heart, open approach	126
Insertion of drug-eluting coronary artery stent(s)	126
Annuloplasty	119
Intraoperative cardiac pacemaker	118
Open and other replacement of mitral valve with tissue graft	110
Initial insertion of dual-chamber device	104
Initial insertion of transvenous leads [electrodes] into atrium and ventricle	101
Venous catheterization for renal dialysis	98
